# Magnetoactive Nanotopography on Hydrogels for Stimulated Cell Adhesion and Differentiation

**DOI:** 10.1002/smsc.202400468

**Published:** 2025-01-27

**Authors:** Md Shariful Islam, Thomas G. Molley, Gagan K. Jalandhra, Jason Fang, Jamie J. Kruzic, Kristopher A. Kilian

**Affiliations:** ^1^ School of Materials Science and Engineering University of New South Wales (UNSW Sydney) Sydney NSW 2052 Australia; ^2^ School of Mechanical and Manufacturing Engineering University of New South Wales (UNSW Sydney) Sydney NSW 2052 Australia; ^3^ School of Chemistry Australian Centre for NanoMedicine University of New South Wales (UNSW Sydney) Sydney NSW 2052 Australia

**Keywords:** biomaterials, hydrogels, nanofibers, stimuli responsive, tissue engineering

## Abstract

Advanced cell culture platforms that vary the biophysical microenvironment are useful tools for mechanobiology studies and for directing the differentiation of adherent cells to therapeutically relevant phenotypes. Herein, the fabrication of magnetoactive nanofiber mats for integration with hydrogels as a platform for dynamic stimulation at the cell–biomaterial interface is demonstrated. Electrospinning is used to form iron oxide‐loaded gelatin‐based nanofibers that are stabilized and cross‐linked to the surface of gelatin methacryloyl hydrogels. The presence of a magnetic field stimulates focal adhesion formation and maturation in adherent adipose‐derived stromal cells, with concurrent changes in cell and nuclear morphology. Adding lineage guiding supplements has been shown to complement biophysical cues, providing optimal conditions for differentiation into osteogenic and adipogenic lineages. The presence of nanofibers at the interface is beneficial to both lineages, but stiffening through an applied magnetic field encourages further osteogenesis while inhibiting adipogenesis. The system is further demonstrated with skeletal myoblasts, where nanotopography and stiffening promote the formation of mature multinucleated muscle cells. This magnetoactive nanofiber platform could prove useful in a wide array of mechanically sensitive cell systems for fundamental studies and for cell production, with flexibility for use with virtually any hydrogel cell culture system.

## Graphical abstract

Magnetic nanofibers are fabricated with gelatin via electrospinning with paramagnetic iron oxide nanoparticles. Nanofiber mats are deposited on the surface of hydrogels of variable stiffness, and the effect of an applied field on the surface structure and hydrogel mechanics is evaluated. Applied fields are found to stimulate focal adhesion and differentiation in adherent adipose‐derived stromal cells.

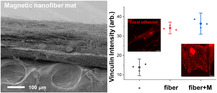

## 1 Introduction

It is widely recognized that cells exhibit high sensitivity to their microenvironment and the composition of living tissues is a complex conglomeration of various cell types integrated within an extracellular matrix (ECM) of numerous proteins and proteoglycans. The ECM exhibits topographical and adhesive characteristics that vary across length scales spanning from the nanometer to the micrometer and is thus reasonable to infer that the topographical attributes of the ECM play a role in regulating cellular activities.^[^ ^1^, ^2^ ^]^ The term “nanotopography” refers to the surface features that arise or are created at the nanoscale level, and numerous functional nanotopographies have been discerned within natural systems due to the presence of pores, ridges, and fibers that exhibit nanometer‐level dimensions. The nanotopography of the ECM can be attributed to a range of intracellular structural features, such as focal adhesion plaques, actin filaments and microtubules, which can be commensurate in size with the nanostructured matrix.^[^ ^3^, ^4^, ^5^ ^]^

Throughout tissue development, cells are stimulated by the numerous and intricately designed bio‐physical (e.g., nanotopography, mechanical properties) and/or biochemical cues present within their respective cellular macromolecular environments.^[^ ^6^ ^]^ Regulation of interactions between cells and their environment, including cell–cell, cell–ECM, cell–soluble factor, and cell–mechanical stimuli interactions, can all occur at the nanoscale level. Researchers have endeavored to gain insights into the interaction between cells and substrates by replicating substratum topographies for in vitro investigations. Contemporary research has brought forth new insight into the response of mammalian cells toward nanoscale features on synthetic surfaces, and several recent scholarly publications have elaborated on this subject matter.^[^ ^2^, ^5^, ^7^ ^]^

In recent years, biophysical cues have become an increasingly favored method for regulating the fate of stem cells.^[^ ^8^ ^]^ Rather than using expensive supplements, the use of biophysical factors at the biomaterial interface has emerged as a promising alternative due to numerous advantages such as lower cost, ease of operation, and higher efficacy in cell differentiation. Implementing biophysical factors in biomaterial development has showcased significant promise for utilization in tissue engineering and regenerative medicine.^[^ ^9^, ^10^ ^]^ Therefore, we aimed to investigate the combined effect of nanotopography paired with compliant substrates that provide dynamic biophysical cues through magnetic activation.

In this study, we report the development of a biomaterial that integrates a layer of nanofibers at the interface of a hydrogel to assess the combined effect of nanotopography and substrate stiffening on adipose‐derived stromal cells (ADSCs) and C2C12 myoblasts. ADSCs, multipotent stem cells from adipose tissue, were chosen for their broad differentiation potential, ease of isolation, and mechanosensitivity, making them ideal for studying the influence of biophysical cues like substrate stiffness and topography on lineage‐specific differentiation. C2C12 myoblasts, a murine skeletal muscle progenitor cell line, were selected for their robust capacity to model myogenesis and evaluate how nanotopography and magnetic stiffening promote muscle differentiation and mature myotube formation. We designed a synthesis procedure to fabricate magneto‐responsive gelatin nanofibers using an electrospinning technique by incorporating iron oxide nanoparticles, followed by a cross‐linking process via heat treatment. The incorporation of magnetic nanoparticles within nanofibers allowed modulation of the nanofibrous interface through an applied magnetic field during cell culture. This magnetic activation provided a dynamic means to influence cellular responses such as spreading, focal adhesion formation, and differentiation.

## 2 Results and Discussion

### 2.1 Synthesis and Characterization of Magnetic Nanofibrous Mats and Nanofiber‐Coated Hydrogel Samples

**Figure 1:**
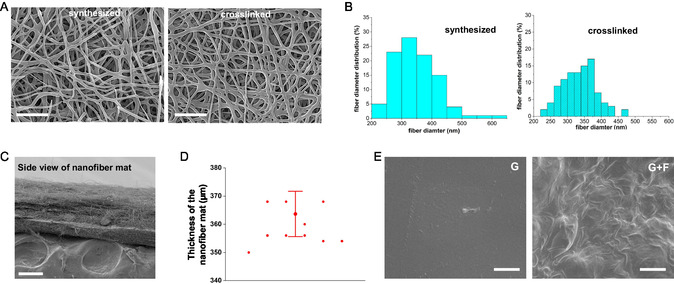
Morphological characteristics of magnetic gelatin nanofibers and nanofiber‐coated hydrogel samples. A) FESEM images of magnetic gelatin nanofibers, displaying a bead‐free morphology. The left panel shows nanofibers in their synthesized form, while the right panel depicts them after the crosslinking process (scale bar: 100 nm), B) distribution of nanofiber diameters before and after crosslinking (*n* = 100), C) side view of the nanofiber mat, highlighting its uniform thickness across the sample (scale bar: 100 μm), D) measurement of nanofiber mat thickness, showing a narrow distribution with most values concentrated around 360 μm, demonstrating the uniformity of the mat (*n* = 3), and E) FESEM micrographs of blank gel (G) and nanofiber‐coated gel (G + F).

We selected gelatin as a base biopolymer for nanofiber and hydrogel fabrication because, as a denatured form of collagen it has native bioactivity for cells.^[^ ^12^ ^]^ Common approaches found in literature to cross‐link gelatin‐based nanofiber were physical cross‐linking (ultraviolet (UV) irradiation^[^ ^13^, ^14^ ^]^ or plasma treatment^[^ ^15^, ^16^ ^]^) and chemical cross‐linking (glutaraldehyde,^[^ ^17^, ^18^ ^]^ EDC/NHS (*N*‐ethyl‐*N*′‐(3‐(dimethylamino)propyl) carbodiimide/*N*‐hydroxysuccinimide,^[^ ^19^, ^20^ ^]^ citric acid,^[^ ^21^, ^22^ ^]^ or polydopamine^[^ ^23^ ^]^). Physical cross‐linking approaches for gelatin nanofibers typically crosslink only the surface of the nanofiber mats,^[^ ^12^ ^]^ and thus requiring further treatment for the fiber interiors. Moreover, physically cross‐linked nanofiber mats often exhibit a gradual reduction in weight subsequent to prolonged immersion in aqueous solutions such as phosphate‐buffered saline (PBS) or cell culture medium.^[^ ^14^ ^]^ In our laboratory, we also found similar results when the nanofiber mats were cross‐linked with UV exposure. While treating with glutaraldehyde was found to be a good approach for cross‐linking gelatin nanofibers, glutaraldehyde is highly toxic^[^ ^24^ ^]^ and may exert cytotoxic environment for cell experiments. Therefore, we identified cross‐linking the synthesized gelatin nanofibers using citric acid and an elevated temperature of 150 °C as the most suitable method for our system, as this approach allowed the cross‐linked nanofibers to completely maintain their fiber morphology after cross‐linking (**Figure** **1**A).

Magnetic gelatin nanofibers were synthesized using an electrospinning method. We mixed magnetic Fe_3_O_4_ nanoparticles with the 25 wt v%^−1^ gelatin solution prior to electrospinning. No discernible beads or clusters of polymer spray were observed in either the synthesized or cross‐linked fibers, demonstrating that the integration of magnetic nanoparticles did not impede the process of fiber synthesis. The average diameter of the synthesized and cross‐linked nanofibers were 348 ± 70 and 334 ± 49 nm, respectively (Figure 1B), indicating that the utilized cross‐linking method did not demolish the inherent fibrous morphology. This observation is consistent with previous findings.^[^ ^21^, ^22^, ^25^ ^]^ A side view of the nanofiber mat (Figure 1B) confirms its uniform thickness, indicating a well‐formed, multilayered structurewith consistency across its cross‐section. Thickness measurements taken in various locations (Figure 1D) further emphasize the uniformity of the mat, with an average thickness of 364 ± 12 μm. We have reported on detailed Fourier transform infrared spectroscopy analysis of the cross‐linking process for similar gelatin nanofibers in our previous paper.^[^ ^26^ ^]^

To evaluate the distribution of the iron oxide nanoparticles, we conducted high‐magnification SEM imaging (Figure S1, Supporting Information). However, due to the nanoscale size of the Fe_3_O_4_ nanoparticles, their direct visualization within the fibers remains challenging. To confirm their incorporation and distribution, EDS mapping was performed, which demonstrates a uniform dispersion of Fe_3_O_4_ nanoparticles throughout the nanofiber mat (Figure S1, Supporting Information). These findings confirm the successful integration of magnetic nanoparticles during fabrication.

The differences in hydrogel surface morphology, with and without the nanofiber layer, are highlighted in Figure 1E. The blank hydrogel (G) exhibits a smooth, uniform surface, typical of a standard hydrogel matrix. In contrast, the nanofiber‐coated hydrogel (G + F) displays a fibrous morphology, clearly indicating the successful integration of the electrospun nanofiber layer.

To test cell adherence, we attached the nanofibers onto glass coverslips using medical grade super glue and cultured 50 000 cells mL^−1^ of ADSCs directly on top of the nanofiber mat. After 24 h of culture, the cells were fixed and stained with phalloidin (for actin filaments) and with 4′,6‐diamidino‐2‐phenylindole (DAPI) (for nuclei). After staining, nanofiber samples were carefully separated from the coverslips for imaging. From Figure S2, Supporting Information, it is evident that cells attached on the nanofiber mat, suggesting that the heat treatment applied to cross‐link the nanofibers did not compromise the gelatin polymer's ability to support cell attachment.

### 2.2 Influence of Nanotopography and Substrate Stiffening in ADSCs Morphology and Differentiation

Changes in the cellular morphology represent an early and discernible indication that can offer essential insights into anticipating cellular activities.^[^ ^27^, ^28^ ^]^ The adhesion of stem cells to the ECM is influenced by the external microenvironment, such as topographic features and mechanical stimuli.^[^ ^29^, ^30^ ^]^ To evaluate the combined effects of nanotopography and substrate stiffness in directing linage specific differentiation of ADSC, we developed a nanofiber‐coated hydrogel system having a layer of magnetic nanofiber mat on top of hydrogels of varying stiffness.

We prepared 8% and 4% gelatin methacryloyl (GelMa) hydrogels to serve as deformable underlying substratum. Figure S3, Supporting Information, depicts the alterations in storage (G′: solid filled shapes) and loss (G″: hollow shapes) modulus over time after the commencement of photo cross‐linking for the GelMa gels. An abrupt elevation in the G′ parameter was noted across the samples within a few min, indicative of the commencement of cross‐linking. After 5 min, the storage modulus (G′) stabilized across the samples yielding a final storage modulus of 0.4 and 10.9 kPa, for the 4% and 8% GelMa hydrogels respectively.

ADSC differentiation is well known to be a mechanosensitive process, with demonstrations of both stiffness^[^ ^31^, ^32^ ^]^ and nanotopography^[^ ^33^, ^34^ ^]^ influencing differentiation, largely through changes in integrin‐mediated adhesion and changes in cell morphology.^[^ ^35^ ^]^ Considering the large increase in cell area and focal adhesion architecture, we reasoned that culturing cells on these nanofibrous interfaces with a deformable underlying substrate would allow dynamic traction force and spreading, thereby influencing the degree of differentiation.^[^ ^35^ ^]^ We chose to examine two mechanosensitive lineages from ADSCs: osteogenesis and adipogenesis. Osteogenic lineage refers to the process that produces osteoblasts, the cells accountable for bone formation. Osteoblasts participate in synthesizing and mineralizing the ECM of bone. On the other hand, adipogenic lineage denotes the cellular differentiation and maturation process leading to the formation of adipocytes, which are accountable for the accumulation and discharge of lipid droplets.^[^ ^36^ ^]^

The influence of nanoscale topographical features on cellular responses such as proliferation, migration, endocytosis, and differentiation has been well established in literature.^[^ ^37^, ^38^, ^39^, ^40^ ^]^ However, what remains less explored is how integrating nanoscale topography, such as the magnetic nanofiber mats developed in this study, can further enhance or direct these lineage‐specific differentiations beyond stiffness alone. The unique combination of nanofibers with tunable stiffness offers a novel platform to investigate how mechanical stimuli and nanoscale topographical features interact to influence cellular behavior. This system allows for more precise control over cell adhesion, morphology, and differentiation processes by offering nanoscale adhesion motifs and the ability to apply magnetic fields for dynamic stiffening. Therefore, in our system, we introduced a layer of nanofiber layer at the interface of the hydrogel substrate, providing nanoscale adhesion motifs to the adhered cells. We explored how this nanotopography influenced the focal adhesion arrangements of the adhered cells and eventually resulting in cell differentiation.

Previous studies have shown that hydrogels with a stiffness greater than 4 kPa are suitable for osteogenic differentiation, while those with a stiffness smaller than 4 kPa are more conducive to adipogenic differentiation.^[^ ^41^, ^42^ ^]^ In this study, we selected 8% GelMa hydrogels with a stiffness of ≈11 kPa as a control group for differentiating ADSCs into osteogenic linage, and 4% GelMa hydrogels with a stiffness of ≈0.4 kPa as a control group for adipogenic linage.

### 2.3 Nanotopography and Substrate Stiffening are Complementary in Driving ADSC Spreading and Focal Adhesion Formation

We cultured 50 000 cells mL^−1^ ADSC on control hydrogels (G) and nanofiber‐coated hydrogels (G + F) made with 8% GelMa solution, both in the presence and absence of static magnets to evaluate the effect of nanotopography in the morphology of ADSC. The cells were fixed after 48 h and stained with phalloidin (cell filaments), DAPI (cell nuclei), and vinculin (focal adhesions), followed by cell morphology assessments.

Integrating nanofiber mats in the hydrogel system significantly augments the proliferation of adipose‐derived stem cells (ADSC) (Figure S4B, Supporting Information). The G + F samples exhibited a 60% increase in ADSC proliferation compared to the control group (G). This number increased slightly (64% in G + F + M samples) when the nanofiber‐coated samples were exposed to the external magnetic field. When comparing the cytoplasmic area of ADSCs cultured on G and G + F samples, no significant difference was observed. However, when nanofiber‐coated hydrogels were exposed to an external magnetic field, the cells demonstrated an 18% increase in their average cytoplasmic surface area (Figure S4A, Supporting Information).

The external magnetic field stiffened the magnetically responsive hydrogel, as evidenced by a 19% increase in Young's modulus in G + F + M samples compared to G + F samples (from 49 ± 0.9 kPa to 58 ± 4.8 kPa, Figure S5C, Supporting Information). This observation is supported by our prior work,^[^ ^26^ ^]^ where a similar magnetic‐responsive hydrogel system demonstrated increased substrate stiffness upon exposure to a magnetic field. Therefore, increases in cell number and cytoplasmic area of ADSCs observed in our study may be attributed not only to the nanotopography provided by the nanofibers but also to the stiffening effect of the magnetic field.^[^ ^43^, ^44^ ^]^ Additionally, we measured surface roughness in the presence and absence of the magnetic field (Figure S5A,B, Supporting Information). The magnetic field reduced the overall roughness of the G + F + M samples by 53% compared to G + F samples (from 2.25 ± 0.3 μm to 1.06 ± 0.2 μm). Notably, earlier studies have reported that reduced nanotopography heights are more favorable for cell spreading,^[^ ^45^ ^]^ which may explain the enhanced cell spreading in G + F + M samples under magnetic field exposure.

**Figure 2:**
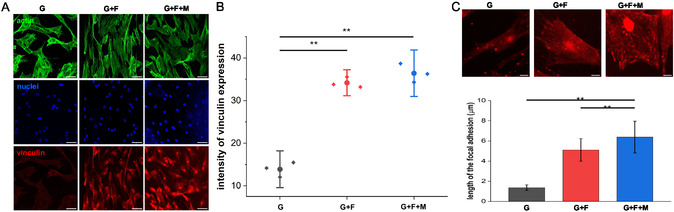
Morphology and proliferation of ADSC seeded on 8% GelMa control and nanofiber‐coated gels. A) Representative confocal images of ADSC, B) intensity of focal adhesion protein, vinculin, expression showing significantly higher expression in nanofiber‐coated gels (scale bar = 100 μm), with **p < 0.01. C) Focal adhesion quantification from vinculin staining (scale bar = 10 μm), *n* = 3, with **p < 0.01.

We then evaluated the adhesive characteristics of ADSC in control (G) and nanofiber‐coated gel samples by immunostaining for the focal adhesion protein vinculin (**Figure** **2**). The results of the study indicate that ADSCs that were cultured on G + F and G + F + M samples showed an increase of 160 and 163% in the vinculin staining intensity, respectively, compared to the control (G). This finding strongly supports the role of nanotopography in promoting the formation of mature focal adhesions. High‐resolution images further revealed the presence of fibrillar focal adhesions in the G + F and G + F + M samples (Figure 2C).

When we measured the focal adhesion lengths, we observed a substantial increase of 272% in G + F and 364% in G + F + M samples compared to the control (G). This significant increase in focal adhesion length in G + F + M, even greater than that in G + F, clearly demonstrates the stiffening effect of the magnetic field. The magnetic field likely enhances the rigidity of the nanofiber‐coated gel, providing a stiffer substrate that facilitates the extension and maturation of focal adhesions. This result is consistent with previous findings, where nanotopography has been shown to significantly influence focal adhesion formation.^[^ ^30^, ^46^, ^47^, ^48^ ^]^ Therefore, in our system, both nanotopography^[^ ^46^, ^48^ ^]^ and substrate stiffening^[^ ^49^, ^50^ ^]^ contributed to the development and maturation of focal adhesions. The inclusion of nanofibers in the nanofiber‐coated hydrogel (G + F) specimens offers nanoscale adhesion motifs and the additional stiffening induced by the magnetic field (G + F + M) further amplified this effect. Taken together, the nanotopography increased ADSC spreading by promoting focal adhesion formation, which in turn encouraged cell proliferation. The further enhancement of cell spreading in the presence of a magnetic field suggests that this platform may be particularly useful for directing stem cell differentiation.

### 2.4 Nanotopography and Substrate Stiffening Influences ADSC Osteogenic Differentiation

The changes in ADSC morphology and enhanced focal adhesion can make cells more susceptible to mechanotransduction and associated processes that converge on pathways of differentiation. To test whether nanotopography and stiffness influence osteogenic lineage specification, we cultured ADSCs on blank gels (G) and nanofiber‐coated gels (G + F) both in proliferation medium and osteogenic differentiation medium for up to 21 days.

**Figure 3:**
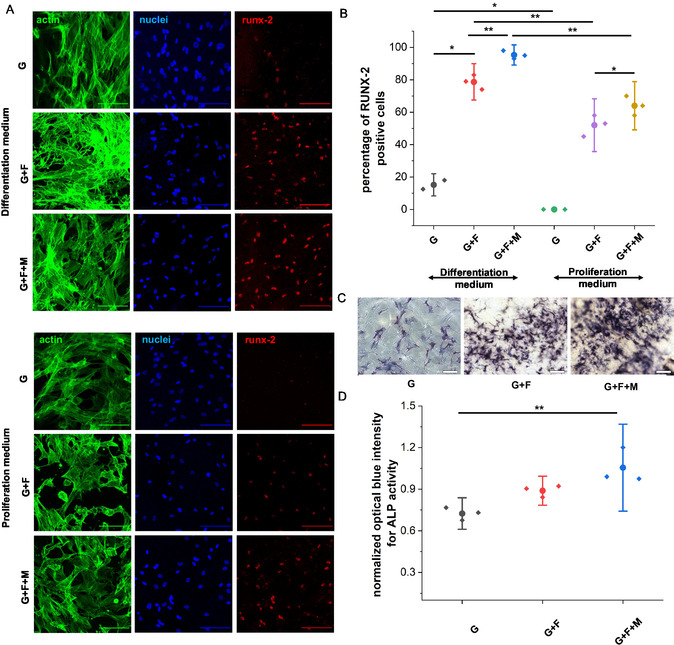
Comparison of ADSCs early‐stage osteogenic differentiation A) representative confocal images of ADSC cells seeded in different 8% GelMa hydrogel samples for 7 days (scale bar:100 μm), B) percentage of cells expressed with RUNX‐2 when cultured in presence of differentiation medium for 7 days, with *p < 0.05 and **p < 0.01, C) representative brightfield microscope images of ADSCs stained for ALP activity when exposed to differentiation medium for 7 days (scale bar:200 μm, *n* = 3), and D) normalized optical blue Intensity of ADSC‐laden 8% GelMa hydrogels after stained with ALP when exposed to differentiation medium for 7 days (*n* = 3), with **p < 0.01.

To evaluate the early‐stage differentiation, cells were fixed and immunostained for the master osteogenesis transcription factor RUNX‐2^[^ ^51^, ^52^ ^]^ after culturing both in proliferation and differentiation medium for 7 days. No discernible RUNX‐2‐positive cells were observed in the blank gel (G) samples exposed to the proliferation medium (**Figure** **3**A). In contrast, only 10% of the cells exhibited positive RUNX‐2 expression when exposed to the differentiation medium. However, the presence of nanofibers (in G + F samples) led to RUNX2 expression in >50% of cells in proliferation medium and >80% of cells in differentiation medium. The magnetic field exposure of the nanofiber‐coated samples (G + F + M) led to RUNX2 expression in >60% of cells in proliferation medium and >95% of cells in differentiation medium (Figure 3B). The presence of the nanofiber mat in the nanofiber‐coated hydrogel samples emulates the bone microenvironment in vivo and can potentially trigger osteogenic differentiation. This result is consistent with previous studies.^[^ ^53^, ^54^, ^55^ ^]^ Furthermore, the magnetic field may increase the interfacial rigidity of the substrate, thereby stimulating differentiation through several complementary biophysical mechanisms.

To supplement the immunofluorescence results, we stained the cells cultured on gel and nanofiber‐coated gel samples with alkaline phosphatase (ALP) after culture in a differentiation medium for 7 days. An upregulation (23% increase) in ALP activity was found in the cells cultured on nanofiber‐coated gel (G + F) samples compared to blank 8% GelMa gel (G) samples (Figure 3C,D). This finding supports the beneficial role of nanotopography in promoting osteogenic differentiation. Furthermore, the cells cultured on the nanofiber‐coated gel (G + F) samples in the presence of a static magnetic field exhibited the highest ALP activity (a 45% increase compared to G). This observation substantiates the notion that substrate stiffening also plays a significant role in enhancing the osteogenic differentiation of ADSCs.

**Figure 4:**
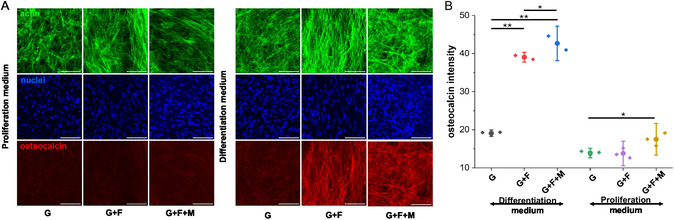
Comparison of late‐stage osteogenic differentiation of ADSC A) representative confocal images of ADSC cells seeded in different 8% GelMa hydrogel samples for 21 days (scale bar = 100 μm), B) plots for osteocalcin intensity in different samples cultured in either differentiation medium or proliferation medium for 21 days (*n* = 3), with *p < 0.05 and **p < 0.01.

After observing empirical evidence in favor of osteogenic lineage specification after 7 days, we next sought to evaluate long‐term osteogenesis through immunostaining after 21 days of culture. ADSCs cultured on the control (G) and nanofiber‐coated 8% GelMa hydrogels (G + F and G + F + M) were stained with osteocalcin, a late‐stage osteogenic differentiation marker.^[^ ^56^, ^57^ ^]^ After 21 days of differentiation, it was observed that ADSC cultured on the nanofiber‐coated samples, both in the absence and presence of the osteogenic medium, exhibited an increased expression of osteocalcin compared to control gels (**Figure** **4**). This finding suggests that the mere existence of nanofiber can trigger ADSC's osteogenic differentiation, a finding that is consistent with prior research.^[^ ^58^ ^]^ When, comparing the osteocalcin intensity of the cells cultured in the presence of osteogenic medium, G + F samples exhibited 105% higher osteocalcin expression than G samples. However, when G + F samples were continuously exposed to a static magnet, a further 9% increase in osteocalcin expression was observed in G + F + M samples than G + F samples (Figure 4B). To supplement the immunostaining data, the mRNA expression levels for osteocalcin were assessed when exposed to an osteogenic medium and found that cells cultured on G + F + M samples exhibited highest mRNA expression (Figure S6, Supporting Information).

Based on all the analyses performed, it can be inferred that the addition of a magnetic nanofiber mat at the interface of the hydrogel (G + F) can significantly enhances the osteogenic differentiation of adipose‐derived stem cells, suggesting the influencial role of nanotopography in promoting osteogenic differentiation. In addition, the use of an external magnetic field to stiffen the substrate can exert an additional positive effect of the osteogenic differentiation of ADSCs. Thus, this study shows how a combination of nanotopography and substrate stiffening can be utilized for directing osteogenic differentiation of ADSCs.

### 2.5 Nanotopography Directs ADSC Adipogenic Differentiation, whereas Substrate Stiffening Blocks it

Upon analyzing the effects of substrate stiffening and nanotopography on the osteogenic differentiation of ADSCs, we examined their influence on adipogenesis. Previous studieshave shown how biophysical cues that direct osteogenesis often negatively regulate adipogenesis.^[^ ^59^, ^60^ ^]^ In contrast, a few studies proved that nanofiber matrices can regulate ADSC's adipogenic differentiation.^[^ ^61^, ^62^ ^]^ Therefore, we introduced a nanofiber mat to the surface of a soft substrate (4% GelMa, 0.4 kPa, Figure S3, Supporting Information) to evaluate the effect of nanotopography and substrate stiffening in ADSC's adipogenic differentiation linage.

We first analyzed the ADSC morphology after 7 days on the control (G) and nanofiber‐coated 4% GelMa hydrogel (G + F) samples and observed that cells cultured in the presence of adipogenic medium across all samples exhibited a comparatively reduced surface area in comparison to those cultured in a proliferation medium (Figure S8A, Supporting Information). This reduction is consistent with the tendency of ADSCs to become smaller and rounder while undergoing adipogenic differentiation.^[^ ^63^ ^]^ Then we examine the average aspect ratios and roundness of cells cultured on G and G + F substrates, in both proliferation and differentiation mediums. Our findings suggest that ADSCs exhibit significantly smaller aspect ratios (with a 14 and 42% reduction in G and G + F, respectively, Figure S8B, Supporting Information) and higher roundness (with a 12 and 90% increment in G and G + F, respectively, Figure S8C, Supporting Information) in the differentiation medium, suggesting these conditions are favorable for adipogenesis. However, when comparing these morphometric changes induced in the samples solely exposed to differentiation medium, it was observed that ADSCs cultured on G + M + F samples displayed smallest roundness and highest aspect ratio (Figure S8B,C, Supporting Information). This observation may suggest that the stiffening of the substrate, caused by magnetic field exposure, could potentially impede adipogenic differentiation. Previous studies also proved that increasing stiffness can affect the adipogenic differentiation potential of ADSCs.^[^ ^59^, ^64^ ^]^

Following an analysis of the morphometric characteristics of the cell cytoplasm, we proceeded to evaluate alterations in nucleus morphometry. It has been recently established that cells undergoing adipogenesis display a reduction in the area and aspect ratio of nuclei, coupled with an increased roundness.^[^ ^65^ ^]^ We did not detect any discernible differences in the average size of the nucleus (Figure S8D, Supporting Information). However, by examining cells cultured on G and G + F samples in differentiation medium, there was a 14% and 19% reduction in nuclei aspect ratio for G and G + F samples, respectively, and a 13 and 22% increase in roundness, compared to cells cultured in proliferation medium (Figure S8E,F, Supporting Information). Moreover, cells cultured in the presence of magnetic field on G + F + M samples demonstrated the smallest nuclei roundness and highest nuclei aspect ratio when comparing among the differentiation medium condition (Figure S8E,F, Supporting Information).

Taken together, the alterations observed in cytoplasm and nuclei morphology are indicative that G and G + F samples provide favorable condition for adipogenesis. However, the biophysical cue of increased substrate stiffness when adding the magnetic field can negate benefit of the preferential nanotopography.

**Figure 5:**
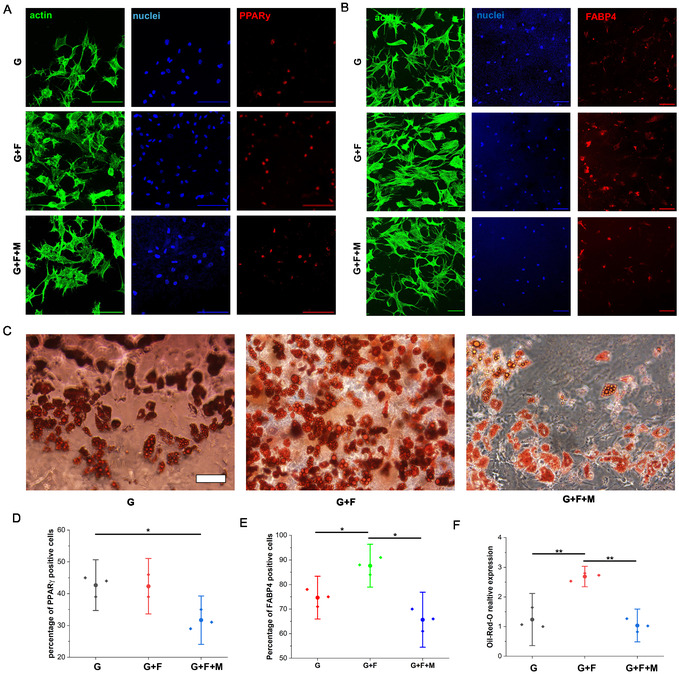
A,B): Confocal images showing ADSCs on nanofiber‐coated 4% GelMa hydrogel samples. (A) Staining for actin (green), nuclei (blue), and PPARγ (red) at 7 days. (B) Staining for actin (green), nuclei (blue), and FABP4 (red) at 21 days (scale bar:100 μm), C) Brightfield images of oil‐red‐o stained cells after 21 days under different conditions, D–F): quantification of adipogenic markers: (D) PPARγ‐positive cells at 7 days, (E) FABP4‐positive cells at 21 days, and (F) oil‐red‐o expression at 21 days, (*n* = 3, with *p < 0.05 and **p < 0.01).

To supplement our morphometric analysis, we immunostained the cells cultured on control (G) and nanofiber‐coated gels (G + F) in both proliferation (Figure S7, Supporting Information) and differentiation medium (**Figure** **5**A) with the master adipogenic regulatory element: peroxisome proliferator‐activated receptor (PPARy).^[^ ^66^ ^]^ As expected, no PPARy expression was observed in the samples exposed to the proliferation medium. In contrast, the data revealed that ≈42% of cells in both the G and G + F samples positively expressed PPARγ when cultured in differentiation medium. Interestingly, in the G + F + M samples, only 28% of cells expressed PPARγ (Figure 5D). Despite extensive study, the evidential support for the beneficial impact of introduction of the magnetic nanofiber mat, i.e., nanotopography on adipogenesis remains inconclusive. Therefore, we examined the differentiation outcome of ADSCs after 21 days. In all samples, ADSCs cultured in differentiation medium exhibited smaller surface area and higher roundness compared to culture in proliferation medium (Figure S9A,C, Supporting Information). Similar trend also observed for nuclei roundness (Figure S9F, Supporting Information). These morphometric changes are indicating that cells may differentiate into adipogenesis. To confirm that, cells were stained with fatty acid‐binding protein‐4 (FABP4) (Figure 5B), which is a late‐stage adipogenic marker under varying conditions.^[^ ^67^ ^]^

We found that 75% of cells cultured on the control 4% GelMa hydrogel (G) and 88% of cells cultured on the nanofiber‐coated gel (G + F) exhibited FABP4 expression (Figure 5E). This result demonstrates how inclusion of the nanofiber mat at the interface of the hydrogel has a marked impact on the adipogenic differentiation of ADSC. However, a 26% decrease in FABP4 expression was noted in the nanofiber‐coated gel samples, subjected to a static magnetic field (G + F + M, Figure 5E). This result indicates that the stiffening of the substrate has a detrimental effect on adipogenic differentiation.

To provide additional evidence for the molecular marker findings, we performed Oil‐Red‐O staining to assess the quantification of lipid droplet formation, a crucial component in cells undergoing adipogenesis. The results showed that the G + F samples had the highest mature lipid droplets (116% more than G, Figure 5F), which is consistent with the immunofluorescent results. Collectively, these findings provide evidence for the beneficial effects of nano‐topographical cues on ADSC adipogenic differentiation. In contrast, stiffening through the application of magnetic fields has been observed to promote cell spreading, reducing the specification of the adipogenic lineage.

### 2.6 Nanotopography Influences Mature Myotube Formation

Skeletal muscle is an important tissue for many regenerative therapies, with evidence for external stimuli guiding differentation, including electrical,^[^ ^68^, ^69^ ^]^ mechanical,^[^ ^70^, ^71^ ^]^ magnetic,^[^ ^72^, ^73^ ^]^ and chemical^[^ ^74^, ^75^ ^]^ approaches. These in vitro maturation techniques provide a compelling alternative to the traditional autologous graft surgery method, aiming to reinstate standard muscular functionality.^[^ ^76^ ^]^ To examine the impact of the nanofiber‐coated hydrogel (G + F) samples on mature myotube formation, we seeded murine myoblast progenitor cells (C2C12) into the control and nanofiber‐coated gels using standard proliferation medium using 8% GelMa gels (≈11 kPa). The cultures were then allowed to proceed for 7 days.

**Figure 6:**
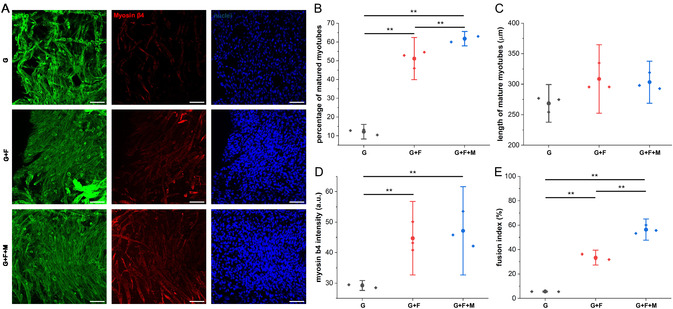
Comparison of C2C12 myogenic differentiation. A) representative confocal images of actin, myosin β4 and nuclei stained C2C12 seeded in different 8% GelMa hydrogel samples over 7 days (scale bar = 100 μm), plots for B) the percentage of mature myotubes present in the samples, C) average length of mature myotubes present in different samples, D) average myosin β4 intensity, and E) fusion index (%) across different samples, (*n* = 3, with *p < 0.05 and **p < 0.01).

During muscle differentiation, a cellular entity comprising three or more nuclei is acknowledged as a mature myotube.^[^ ^77^, ^78^ ^]^ Most cells in nanofiber‐coated hydrogel samples, irrespective of the presence or absence of a magnetic field, displayed a morphology characterized by multiple nuclei. In contrast, the number of cells exhibiting this morphology was relatively low in control gels (**Figure** **6**). While examining the number of mature myotubes present in these samples after 7 days, it was observed that the G + F samples demonstrated a 4.3‐fold greater number of mature myotubes than the control gel samples (Figure 6B). The possible reason could be the presence of nanofiber in the nanofiber‐coated hydrogel samples that offer high specific surface area and suitable topographical cue for the C2C12 cells to form mature myotubes.^[^ ^76^ ^]^ When the G + F samples were continuously exposed to the static magnetic field a further increase in mature myotubes formation was observed (51% and 61% in G + F and G + F + M samples, respectively, Figure 6B). Previous studies have demonstrated that substrate stiffening can accelerate mature myotube formation,^[^ ^71^, ^79^, ^80^ ^]^ and our findings align with this concept. The magnetic field likely enhances the stiffness of the nanofiber‐coated hydrogels, contributing to the observed increase in myotube formation in G + F + M samples. To support the maturation status of these cells, we did the morphometric analysis and found highest fusion index (Figure 6E) and myotube length (Figure 6C) in the G + F + M samples.

Next, we examined the relation of these morphometric changes with myogenic differentiation of C2C12. Upon comparing the control (G) and nanofiber‐coated (G + F) gel samples made from 8% GelMa solution, a notable increase of sixfold and tenfold in the expression of myosin β4 was observed in the G + F and G + F + M samples, respectively, in comparison to the control gel (Figure 6D). These quantitative findings demonstrate that nanotopography plays a key role in promoting myogenic differentiation of C2C12 cells, with magnetic stiffening providing additional support for their maturation.

## 3 Conclusion

In this manuscript, we demonstrated the fabrication of an iron oxide nanoparticle‐loaded magnetic gelatin nanofiber mat for deposition onto cell culture substrates. These magnetic nanofiber mats were incorporated onto the surface of GelMa hydrogels to create a novel nanofiber‐coated hydrogel system with both nanotopography and tunable stiffness, controlled by applying a magnetic field. The nanofiber‐coated gels showed significant potential in directing ADSC differentiation into various lineages, driven by the combination of nanotopographical features and magnetic field‐induced interfacial substrate stiffening. Our findings highlight how the combined effects of nanotopography and magnetic actuation‐induced stiffness significantly enhance ADSC osteogenic differentiation while inhibiting adipogenesis. The versatility of these hydrogels was further demonstrated by their ability to promote myogenic differentiation in the C2C12 model, with enhanced mature myotube formation observed under magnetic stimulation. Therefore, this stimulus‐responsive nanofiber‐coated hydrogel system offers excellent potential as a platform for mechanobiology studies and for in vitro differentiation and maturation strategies for regenerative therapies.

## 4 Experimental Section

4.1

4.1.1

##### Synthesis of Magnetic Nanofibrous Mats

To synthesize the magnetic nanofibrous mats, a solution of 25 wt v%^−1^ Type‐A gelatin (Sigma‐Aldrich Pty Ltd. solution, G2500) in 90% acetic acid (Chem‐Supply Pty Ltd., AA009) was prepared by stirring at 50 °C for 4 h. Subsequently, citric acid (Chem‐Supply Pty. Ltd., CA014) and sodium hypophosphite (Chem‐Supply Pty. Ltd., 10039562), corresponding to 15% and 7.5% of gelatin's dry weight, respectively, were added and the mixture was stirred for an additional 2 h. Following this, 5 wt v%^−1^ Fe_3_O_4_ nanoparticles (50–100 nm, Sigma‐Aldrich, 637106) were incorporated and uniformly dispersed using probe sonication (Qsonica Q500, USA) at 20% amplitude for 1 min. Electrospinning was performed using an electrospinning machine (Bioinicia Ltd., Spain) at 25 kV, maintaining a needle‐to‐collector distance of 15 cm and a flow rate of 2 μL min^−1^, with fibers being collected on aluminum foil. The fibers were subsequently cross‐linked by heating at 150 °C for 4 h in a vacuum oven. The morphology of the nanofibers and the thickness of the nanofibrous mats were characterized using field emission scanning electron microscopy (FESEM, FEI Nova NanoSEM 230).

##### Synthesis and Characterization of Nanofiber‐Coated Hydrogel

Following the cross‐linking process of the nanofibers, the resulting nanofiber mat was manually removed from the aluminum foil and trimmed with scissors to achieve an approximate area of 25 mm^2^. The nanofibers were sterilized with UV light for 30 min. To produce the nanofiber‐coated hydrogel, a solution of gelatin methacryloyl (GelMa) was dispensed onto a 3D‐printed plastic mold (6 × 6 × 2.5 mm^3^) placed on a glass slide. Carefully, a layer of nanofiber was added on top of the solution. The fiber mat was soaked for 30 s, then cross‐linking was initiated. Cross‐linking was accomplished by exposing the sample to a 395 nm UV light (purchased from BigW, product no; 9900086793), which was positioned 5 cm above it. The exposure lasted for 1 min. Hydrogel samples were categorized as follows: blank hydrogels without nanofibers (G), nanofiber‐coated hydrogels (G + F), and nanofiber‐coated hydrogels exposed to a magnetic field (G + F + M).

To analyze the morphology of the hydrogel samples, freeze‐dried specimens were imaged using an FESEM. Changes in surface roughness were evaluated using a 3D laser scanning microscope (VK‐X200). To measure roughness and Young's modulus under the influence of a magnetic field, the same magnets used during cell culture experiments were placed beneath the nanofiber‐coated samples to replicate experimental conditions.

The Young's modulus of the samples was determined using an Anton Paar MCR 302 Rheometer. Compression tests were performed with the samples compressed to 20% strain. The modulus was calculated from data in the 10–15% strain region. These tests were conducted with and without the magnetic field to evaluate its influence on the mechanical properties of the hydrogel samples.

##### Rheological Analysis

Rheological measurements were conducted using an Anton Paar MCR 302 Rheometer, using a parallel plate geometry of 25 mm disk and 1 mm measuring distance, with 600 μL of the gel solution. The 1 mm thickness was selected to ensure uniform contact between the plate and the sample, and the sample volume ensured complete surface coverage. This study employed oscillatory measurements using a 0.02% strain and a 1 Hz frequency during the gelation process at a temperature of 20 °C. The parameters were chosen based on preliminary tests to remain within the linear viscoelastic regime, where the gel structure remains intact. The gel solution was prepared at 37 °C to maintain its liquid state and immediately transferred to the rheometer to prevent premature gelation. Measurements were conducted at 20 °C, a temperature chosen to match the room temperature conditions under which the samples were prepared for cell culture studies, ensuring consistency. In situ UV cross‐linking was achieved using a 395 nm UV light positioned 5 cm below the quartz crystal stage for 60 s, ensuring uniform illumination and consistent cross‐linking.

##### Cell Culture

To expand ADSC and C2C12 cells, low‐glucose Dulbecco's modified Eagle's medium (DMEM) was used for ADSCs, and high‐glucose DMEM was used for C2C12 cells. The ADSC culture medium was supplemented with 10% fetal bovine serum (FBS) and 1% penicillin‐streptomycin, while the C2C12 culture medium was supplemented with 15% FBS and 1% penicillin‐streptomycin. Both cell types were incubated at 37 °C with 5% CO_2_ in an incubator, with the culture medium refreshed every 48 h. ADSC cells were passaged when they reached 80–85% confluency, and C2C12 cells were passaged at 70–75% confluency. Cells from passages 4–10 were used in all experiments.

For all samples, 50 000 mL^−1^ cells were seeded on the hydrogel samples and 500 μL of either proliferation medium or differentiation medium was used. Medium changes were executed every 48 h. This seeding density was determined based on prior trial‐and‐error experiments in our lab, which aimed to identify an optimal density for achieving robust cell adhesion, proliferation, and differentiation on the hydrogel surface. This density provided a balance between sufficient cell coverage for effective interaction with the substrate and avoiding overcrowding that could hinder nutrient availability.

For the ADSC differentiation study, medium was first changed after 48 h and replaced with the differentiation medium. StemPro Osteogenic differentiation media (Thermo Fisher Scientific, A1007201) and StemPro Adipogenic differentiation media (Thermo Fisher Scientific, A1007001) were used as differentiation medium. High‐glucose DMEM (ThermoFisher Scientific, 11965092) was used for both the expansion and differentiation of C2C12 cells.

To induce a static magnetic field, a neodymium block magnet measuring 25 × 12.5 × 6 mm^3^ and capable of exerting a magnetic flux of 0.36 T (purchased from: aussiemagnets.com.au) was positioned beneath each of the samples.

##### Histological Chemical Staining of Nanofiber‐Coated Hydrogel Samples

All samples were fixed with 4% paraformaldehyde at room temperature for 30 min before histo‐chemical staining. This study utilized ALP and Oil‐Red‐O staining techniques to visualize osteogenic and adipogenic differentiation, respectively.

ALP staining was conducted using a tablet (Sigma Aldrich, B5655). Each tablet was dissolved in 10 mL of deionized water by a tube vortex mixture for 10 min. Fixed gels were soaked in BCIP/NBT solution for 1 h and washed three times with deionized water before imaging by a bright field microscope.

Oil‐Red‐O powders were first dissolved in 100% isopropyl alcohol at 3 mg mL^−1^ concentration by a tube vortex mixture for 20 min and used as a stock solution. A mixture of stock solution and deionized water (60:40, stock solution: deionized water) filtered by a 0.45 μm filter was finally used as a working solution. Before soaking the samples in the Oil‐Red‐O working solution, gels were soaked for 20 min in 60% Isopropyl alcohol in deionized water. After removing the 60% isopropyl alcohol solution, samples were soaked in a working solution for 60 min at room temperature.

All samples were washed three times before imaging by a brightfield microscope with a 20× objective.

##### Immunostaining of Nanofiber‐Coated Hydrogel Samples

Hydrogel samples with cells were fixed with 4% paraformaldehyde. After fixing, samples were washed twice with PBS. The samples were permeabilized and blocked by soaking them in 0.5 wt% Triton X‐100 solution for 30 min and then washing them three times in PBS with 1% bovine serum albumin (BSA) On a petri dish, primary and secondary antibody labeling was done by flipping the samples onto the antibody solution for 1 h. Before secondary antibody labeling, samples were washed thrice with 1% BSA in PBS. RUNX‐2 (Abcam Australia, ab76956), osteocalcin (Thermo Fisher, PA5‐96529), peroxisome proliferator‐activated receptor (PPARy, Cell Signaling, C26h12), fatty acid‐binding protein‐4 (FABP4, Abcam Australia, ab92501), and myosin β4 (Thermo Fisher, 14650382) were used as primary antibodies. Before imaging, samples were cleared with cubic‐2 clearing solution for 24 h.

The cubic‐2 solution was prepared by slightly modifying the protocol described in the literature.^[^ ^11^ ^]^ A mixture of 50 w v^−1^% sucrose (Sigma Aldrich, 57‐50‐1) and 25 w v^−1^% urea (Sigma Aldrich, 57‐13‐6) was dissolved in deionized water by stirring. When the mixture temperature reached 60 °C, 10 w v^−1^% triethanolamine was added and stirring was continued for 4 h. Cubic solution was stored at room temperature for up to 4 weeks.

Imaging of the samples was carried out using Zeiss LSM 800 microscope with either a 10× or 20× objective.

Average cell cytoplasm and nucleus areas, aspect ratio, and roundness of ADSCs were determined by analyzing the actin cytoskeleton with phalloidin staining and nucleus with DAPI staining, respectively, using ImageJ software. Specifically, 100 cells were examined using ImageJ from three replicates. The percentage of RUNX‐2 and PPARy/FABP4‐positive cells was calculated by dividing the RUNX‐2‐ and FABP4‐positive cells by the total number of cells. To determine the level of osteocalcin expression by the ADSCs after 21 days, the raw intensity of cells that expressed osteocalcin was measured using Image J software after subtracting the background signal.

The average length of the myotubes was measured from three replicates, and all the myotubes in the samples were counted. To assess the relative expression levels of myosin β4 in the cultured cells for 7 days, the myosin β4 image channels were separated, and the intensity was measured using ImageJ software. Furthermore, the fold‐increase levels of myosin β4 were determined by normalizing the intensity level of each group with respect to that of the blank hydrogel at day 7. The fusion index was calculated by determining the total nuclei incorporated into myotubes compared to the total number of nuclei in the image.

##### Real‐Time Polymerase Chain Reaction Analysis

Hydrogel samples with ADSC cultured for 21 days in differentiation medium were first rinsed with PBS and allowed to incubate in a cell culture incubator for 30 m to remove excess media. A RNeasy mini kit (Qiagen, 74104) was used to extract ribonucleic acid (RNA) from the cell‐laden hydrogels according to the kit's instructions. A high‐capacity complementary deoxyribonucleic acid (cDNA) reverse transcription kit (Applied Biosystems, 4368814) was then used to prepare cDNA, which was added to TaqMan real‐time polymerase chain reaction (RT‐PCR) Master mix (Applied Biosystems, 4304437) with necessary primers and loaded into wells of a 96‐well plate. QuantStudio 7 Flex RT‐PCR System was used to run the qPCR program, and the software generated automatic cycle threshold (CT) values per replicate, which were converted into ΔΔCT values using glyceraldehyde 3‐phosphate dehydrogenase (GAPDH) as an internal control. The primers used included GAPDH (Hs01587814_g1) and bone gamma‐carboxyglutamic acid‐containing protein (Hs02786624_g1), a key marker of osteogenic differentiation.

##### Statistical Analysis

Statistical analysis was performed using one‐way analysis of variance followed by Tukey's post‐hoc honestly significant difference test to evaluate differences between groups. A *P*‐value of less than 0.05 was considered to indicate statistical significance. Symbols used to represent significance levels in the figures include: * for *P* < 0.05, ** for *P* < 0.01, and *** for *P* < 0.001. Whiskers in interval and box plots represent the standard deviation.

## Conflict of Interest

The authors declare no conflict of interest.

## Author Contributions

**Kristopher A. Kilian**: conceptualization: (lead); investigation: (equal); methodology: (equal); project administration: (lead); resources: (lead); and supervision: (lead). **Md Shariful Islam**: formal analysis: (lead); investigation: (equal); validation: (lead); visualization: (lead); and writing—original draft: (lead). **Thomas G. Molley**: formal analysis: (supporting); investigation: (supporting); and writing—review and editing: (supporting). **Gagan K. Jalandhra**: investigation: (supporting) and visualization: (supporting). **Jason Fang**: investigation: (supporting) and methodology: (supporting). **Jamie J. Kruzic**: conceptualization: (supporting); investigation: (supporting); methodology: (supporting); and supervision: (supporting).

## Supporting information

Supplementary Material

## Data Availability

The data that support the findings of this study are available from the corresponding author upon reasonable request.
