# Supplementary material for: Magnetoactive Nanotopography on Hydrogels for Stimulated Cell Adhesion and Differentiation
Source: Small Sci. 2025 Jan 27;5(4):2400468. doi: 10.1002/smsc.202400468 (PMC12244508; doi:10.1002/smsc.202400468)
Supplement: Supplementary file 1 — Supplementary Material [file SMSC-5-2400468-s001.pdf]

MAGNETOACTIVE NANOTOPOGRAPHY ON HYDROGELS FOR STIMULATED CELL ADHESION AND DIFFERENTIATION

Md Shariful Islam[1], Thomas G. Molley[1], Gagan K. Jalandhra[1], Jason Fang[1], Jamie J. Kruzic[3], Kristopher A. Kilian[1,2]*

[1]School of Materials Science and Engineering, University of New South Wales (UNSW Sydney), Sydney NSW 2052, Australia

[2]School of Chemistry, Australian Centre for NanoMedicine, University of New South Wales (UNSW Sydney), Sydney NSW 2052, Australia

[3]School of Mechanical and Manufacturing Engineering, University of New South Wales (UNSW Sydney), Sydney NSW 2052, Australia

*corresponding author k.kilian@unsw.edu.au

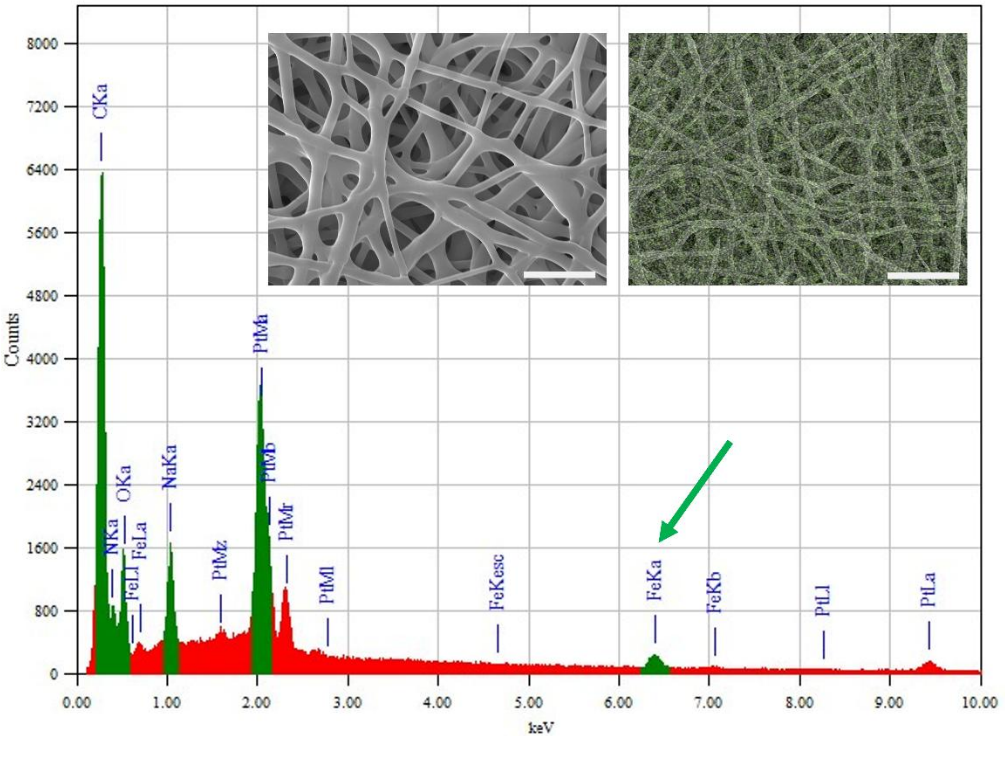

Figure S1: SEM and EDS analysis of electrospun nanofibers containing Fe_3O_4 nanoparticles. The top left panel shows a high-magnification SEM image of the electrospun nanofiber mat, highlighting the uniform and bead-free morphology of the fibers. The top right panel presents EDS mapping of the nanofiber mat, confirming the homogeneous distribution of Fe_3O_4 nanoparticles throughout the structure

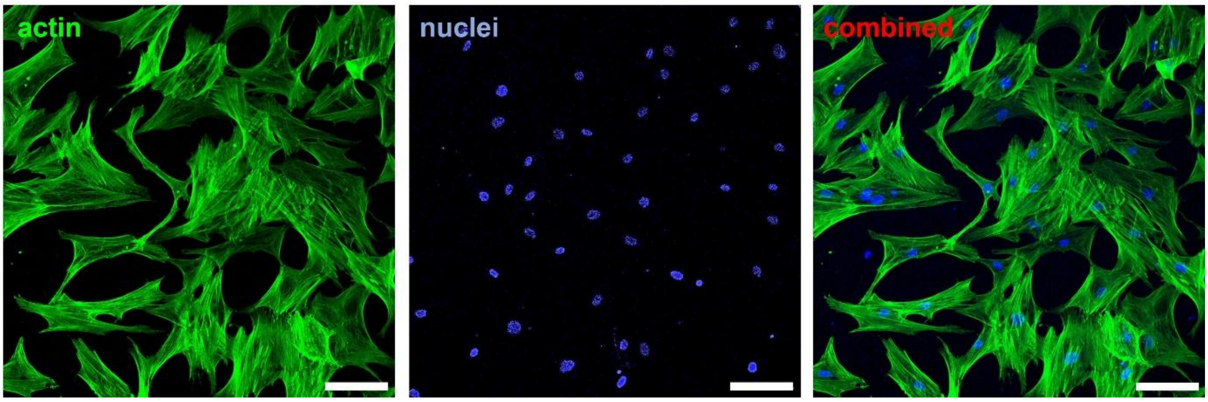

Figure S2: Representative confocal microscope images of adipose-derived stem cells (ADSCs) on crosslinked nanofiber scaffolds after 24 hr. Actin filaments (green) show prominent stress fibers, while DAPI-stained nuclei (blue) illustrate cell distribution (scale bar: 100 µm).

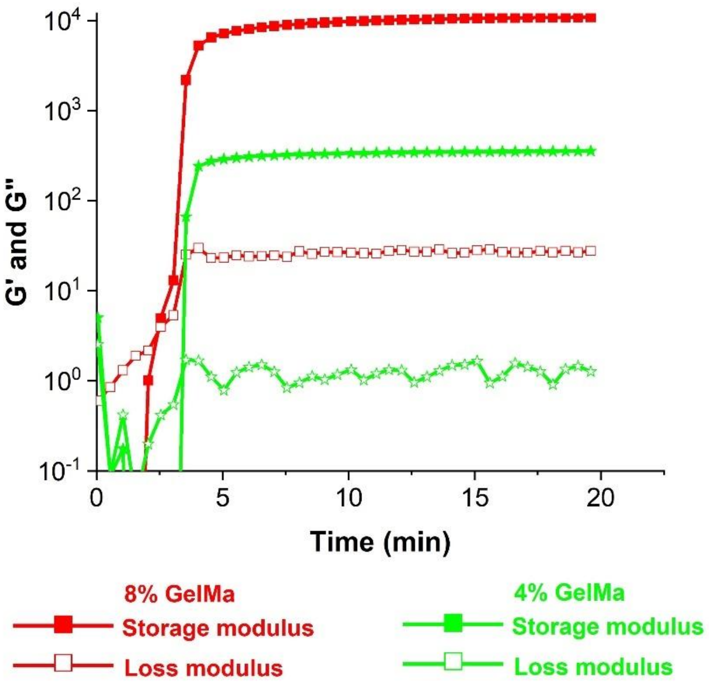

Figure S3: Rheological analysis of 4% and 8% GelMA hydrogels, illustrating the evolution of storage modulus (G') and loss modulus (G'') over time under 1 Hz frequency and 0.2% strain. Both hydrogels demonstrate an initial rapid gelation phase, with the 8% GelMA showing a higher G' and G'' than the 4% GelMA. The data capture the viscoelastic properties and stability of the hydrogels over 20-min.

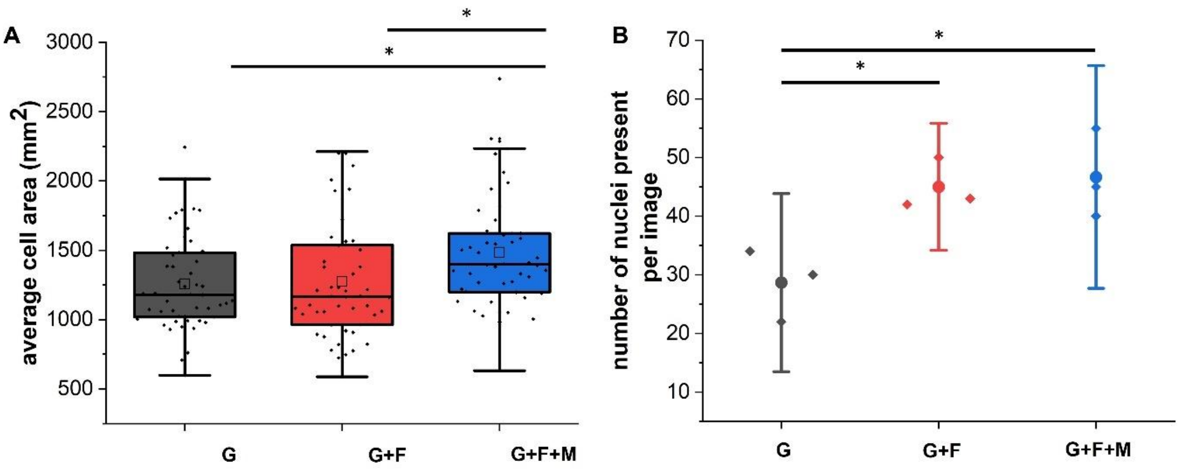

Figure S4: A. Quantitative analysis of cell morphology and proliferation on different substrates after 48 hr. (A) Box plot comparison of the average cell cytoplasmic area showing significantly higher surface areas on nanofiber-coated GelMA samples (G+F and G+F+M groups) compared to GelMA alone (G). (B) Comparison of the number of nuclei per image, indicating increased cell proliferation on nanofiber-coated GelMA (G+F+M group) relative to GelMA alone (G) and GelMA with nanofibers (G+F). Data are presented as mean ± SD, with statistical significance marked by * ($p < 0.05$)

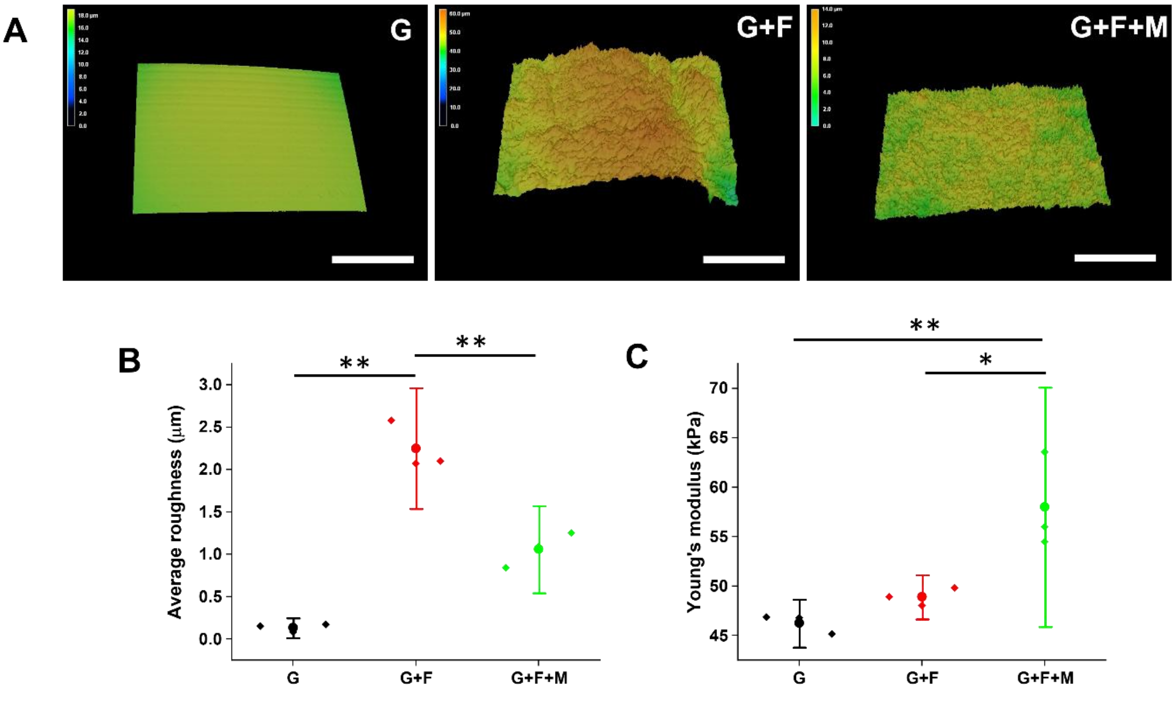

Figure S5: Characterization of surface roughness and mechanical properties of hydrogel samples. (A) 3D laser microscopy image depicting the overall

average surface roughness of the samples. (B) Quantitative analysis of surface roughness, showing significantly higher roughness in the nanofiber-coated samples compared to the blank hydrogel (G), and (C) Young's modulus measurements obtained using a rheometer, demonstrating increased mechanical stiffness in the nanofiber-coated samples under the influence of a magnetic field (G+F+M), (n=3, with *p<0.05 and **p<0.01).

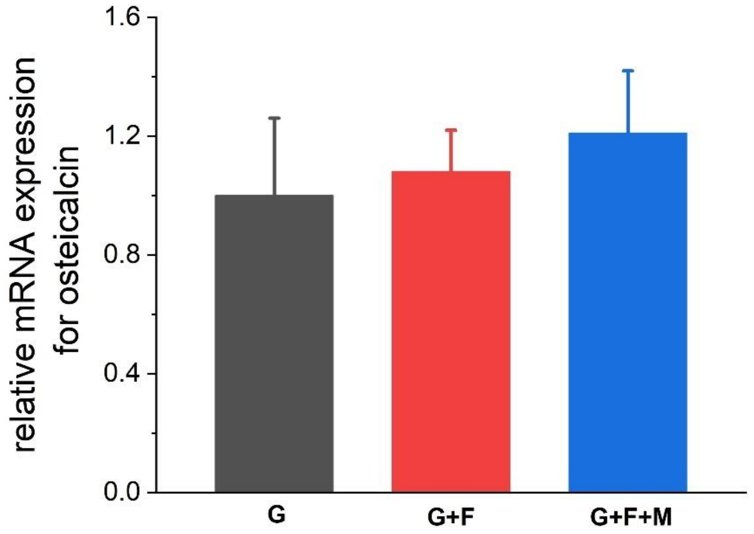

Figure S6: Relative mRNA expression levels of osteocalcin in adipose-derived stem cells (ADSCs) cultured in osteogenic medium for 21 days. The data are normalized to the expression levels in cells cultured on GelMA alone (G) and show a slight increase in osteocalcin expression in cells cultured on nanofiber-coated GelMA (G+F and G+F+M), suggesting enhanced osteogenic differentiation.

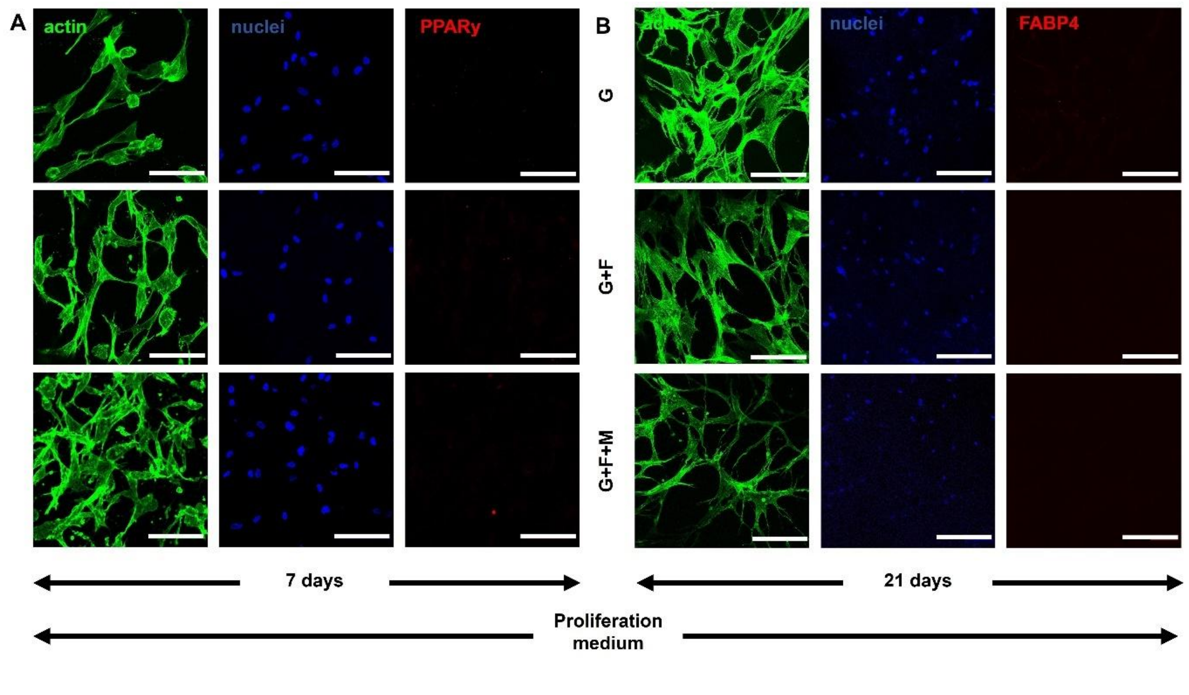

Figure S7: Representative confocal micrographs of adipose-derived stem cells (ADSCs) cultured in proliferation medium on nanofiber-coated hydrogel samples over (A) 7 days and (B) 21 days. Actin cytoskeleton (green), nuclei (blue), and adipogenic markers PPARγ (red in A) and FABP4 (red in B) were stained to assess cell morphology and differentiation potential. Minimal expression of PPARγ and FABP4 indicates limited adipogenic differentiation under proliferation conditions, while actin staining highlights the well-spread morphology of the ADSCs on all sample types (scale bar: 100 μm).

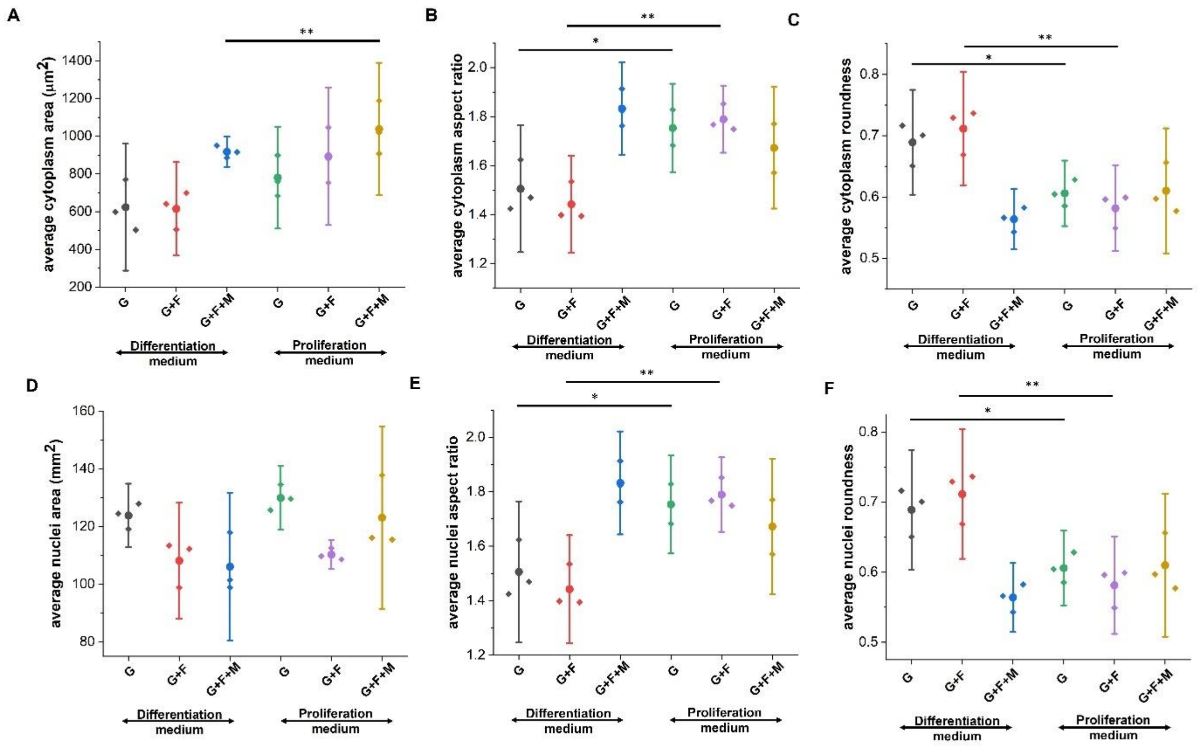

Figure S8: Morphometric analysis of adipose-derived stem cells (ADSCs) cultured for 7 days on different nanofiber-coated hydrogel samples in differentiation and proliferation media. (A-C) Cytoplasmic morphometric parameters: (A) average cytoplasmic area, (B) aspect ratio, and (C) roundness, highlighting significant differences in cell morphology across different substrates and media conditions. (D-F) Nuclear morphometric parameters: (D) average nuclear area, (E) aspect ratio, and (F) roundness, showing the influence of substrate and media on nuclear morphology. Data are represented as mean ± SD with significant differences marked by * ($p < 0.05$) and ** ($p < 0.01$).

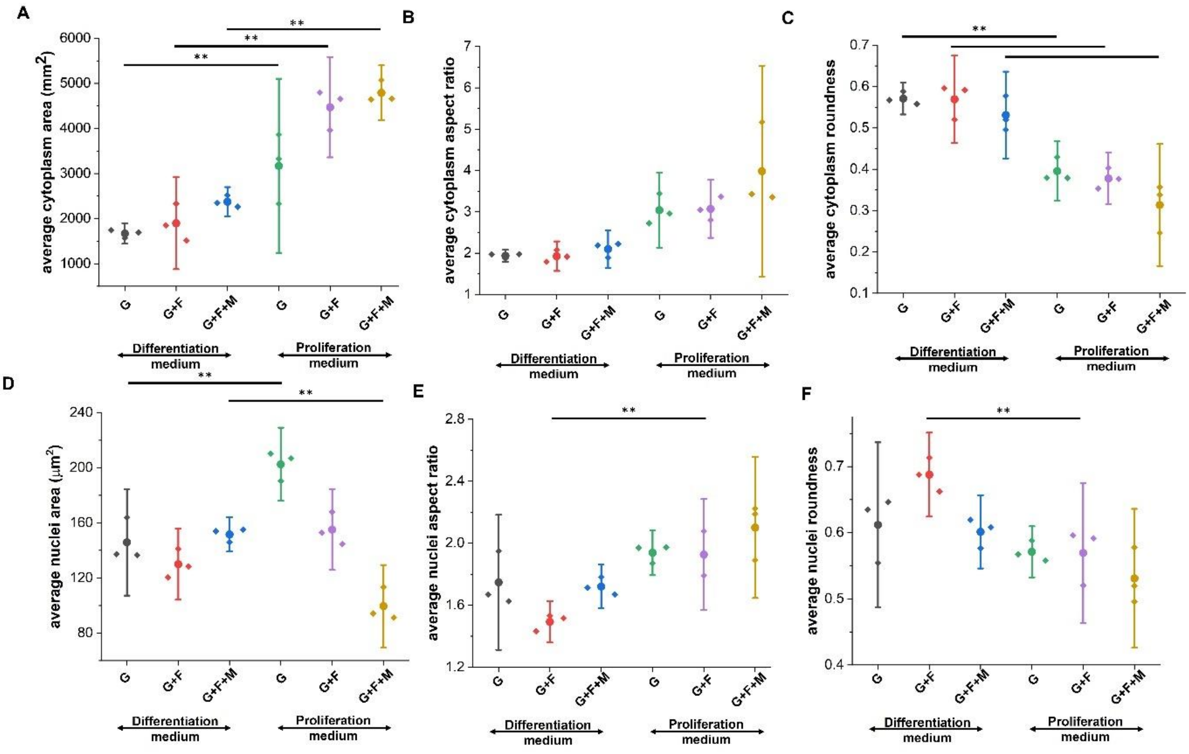

Figure S9: Morphometric analysis of adipose-derived stem cells (ADSCs) cultured for 21 days on nanofiber-coated hydrogel samples in differentiation and proliferation media. (A-C) Cytoplasmic morphometric parameters: (A) average area, (B) aspect ratio, and (C) roundness. (D-F) Nuclear morphometric parameters: (D) average area, (E) aspect ratio, and (F) roundness. The data illustrate significant differences in cell and nuclear morphology across the different substrates and media conditions after 21 days of culture. Significant differences between groups are indicated by ** ($p < 0.01$).
